# The Role of Organelle Stresses in Diabetes Mellitus and Obesity: Implication for Treatment

**DOI:** 10.1155/2015/972891

**Published:** 2015-11-03

**Authors:** Yi-Cheng Chang, Siow-Wey Hee, Meng-Lun Hsieh, Yung-Ming Jeng, Lee-Ming Chuang

**Affiliations:** ^1^Graduate Institute of Medical Genomics and Proteomics, National Taiwan University, Taipei 100, Taiwan; ^2^Department of Internal Medicine and Center for Obesity, Lifestyle and Metabolic Surgery, National Taiwan University Hospital, Taipei 100, Taiwan; ^3^Institute of Biomedical Science, Academia Sinica, Taipei 100, Taiwan; ^4^Graduate Institute of Pathology, National Taiwan University, Taipei 100, Taiwan; ^5^Department of Pathology, National Taiwan University Hospital, Taipei 100, Taiwan; ^6^College of Medicine, National Taiwan University, Taipei 100, Taiwan; ^7^Institute of Preventive Medicine, College of Public Health, National Taiwan University, Taipei 100, Taiwan

## Abstract

The type 2 diabetes pandemic in recent decades is a huge global health threat. This pandemic is primarily attributed to the surplus of nutrients and the increased prevalence of obesity worldwide. In contrast, calorie restriction and weight reduction can drastically prevent type 2 diabetes, indicating a central role of nutrient excess in the development of diabetes. Recently, the molecular links between excessive nutrients, organelle stress, and development of metabolic disease have been extensively studied. Specifically, excessive nutrients trigger endoplasmic reticulum stress and increase the production of mitochondrial reactive oxygen species, leading to activation of stress signaling pathway, inflammatory response, lipogenesis, and pancreatic beta-cell death. Autophagy is required for clearance of hepatic lipid clearance, alleviation of pancreatic beta-cell stress, and white adipocyte differentiation. ROS scavengers, chemical chaperones, and autophagy activators have demonstrated promising effects for the treatment of insulin resistance and diabetes in preclinical models. Further results from clinical trials are eagerly awaited.

## 1. Introduction

*Type 2 Diabetes Mellitus and Obesity: The Role of Nutrient Oversupply*. Type 2 diabetes mellitus (T2DM) has become a global pandemic with huge health impact in recent decades. T2DM is a chronic progressive disorder characterized by peripheral insulin resistance in skeletal muscle, liver, and adipose tissue and the failure of pancreatic beta-cells to compensate for peripheral insulin resistance. Peripheral insulin resistance usually appears before the onset of hyperglycemia. Attenuated insulin action leads to reduced glucose uptake in skeletal muscle, reduced glucose uptake and increased lipolysis in adipose tissue, and decreased glycogen synthesis and increased glucose output of the liver, resulting in elevated plasma glucose and fatty acid levels [1]. To compensate for peripheral insulin resistance, pancreatic *β*-cells, which constitute only ~1% of pancreatic mass, have to dramatically increase proinsulin synthesis, imposing heavy biosynthesis burden on *β*-cells. Ultimately, pancreatic *β*-cells fail to overcome the resistance and frank hyperglycemia develops.

Obesity is the major driver of insulin resistance and T2DM. Obesity results from chronic imbalance of energy intake in excess of energy expenditure. Large prospective studies showed that lifestyle modification including diet restriction and exercise prevented the progression from prediabetes to diabetes by ~60% [2, 3]. In rhesus monkeys, long-term caloric-restricted diet drastically reduces incident diabetes or prediabetes [4]. These data clearly demonstrate excessive nutrient is critical for the development of obesity, leading to insulin resistance and T2DM.

*Molecular Mechanism of Insulin Resistance*. The molecular mechanism of insulin resistance is still not fully elucidated. Binding of insulin to insulin receptor triggers tyrosine autophosphorylation of the insulin receptor, which in turn phosphorylates the adaptor proteins insulin receptor substrate (IRS) proteins on tyrosine residues [5]. Tyrosine-phosphorylated IRS proteins recruit phosphoinositide-3-kinase (PI3K), a heterodimer consisting of a regulatory subunit p85 and a tightly associated catalytic subunit p110. Binding of the p85 regulatory subunit to phosphorylated IRS relieves catalytic subunit p110 and initiates a complex of signaling cascades that mediates downstream insulin action.

IRS proteins harbor several serine/threonine phosphorylation sites, which served as negative regulatory nodes that block insulin signaling triggered by tyrosine phosphorylation [6]. Several serine/threonine kinases including the cellular nutrient sensor mammalian target of rapamycin (mTOR) and ribosomal S6 kinase 1 (S6K1), the stress mediators c-Jun NH2-terminal kinases (JNK), and the proinflammatory I*κ*B kinase *β* (IKK*β*) and protein kinase *θ* (PKC-*θ*) block insulin signaling by serine-phosphorylation of IRS [6].

## 2. The Role of Endoplasmic Reticulum Stress and Unfolded Protein Response (UPR) in Diabetes and Obesity

The ER is a specialized organelle essential for synthesis and folding of secreted and ER-resident proteins, maintenance of intracellular calcium homeostasis, and lipid synthesis. The protein concentration in ER lumen is very high. Therefore, increased demand for protein synthesis or accumulation of misfolded protein in the ER luminal causes “ER stress,” which triggers conserved transcriptional and translation programs, termed unfolded protein response (UPR), to cope with the ER stress [7]. The UPR are mediated by three ER membrane-bound mediators including inositol-requiring enzyme-1 (IRE-1), PKR-like endoplasmic reticulum kinase (PERK), and activating transcription factor 6 (ATF6), which are bound by the abundant ER chaperones glucose-regulated protein 78 (GRP78) in unstressed conditions. In stressful conditions when misfolded proteins accumulated, GRP78 chaperones are sequestered by misfolded proteins, releasing these UPR mediators. IRE1, an ancient ribonuclease and the oldest branch of UPR, cleaves 26-bp segment from the mRNA of x-box binding-1 (*XBP-1*) gene, creating an active/splice form of XBP-1 (XBP-1s). XBP-1s launches transcriptional programs to increase chaperone production, membrane biosynthesis, and gradation of misfolded proteins. Release of ATF6 from ER membrane unmasks its Golgi localization sequence. After processing by two proteases in Golgi, ATF6 is translocated to the nucleus to regulate the expression of genes encoding chaperones, enzymes for protein degradation, and ER membrane biogenesis. The release of PERK form membrane leads to its oligomerization and autophosphorylation. Activated PERK phosphorylates the eukaryotic initiation factor 2*α* (eIF2*α*), thereby suppressing general mRNA translation. However, specific mRNAs are preferentially translated when eIF2*α* is inhibited, including the transcriptional factor ATF4. Two downstream genes of ATF4 are the proapoptotic transcription factor C/EBP homologous protein (CHOP) and the growth arrest and DNA damage–inducible 34 (GADD34) which counteracts PERK's action by dephosphorylating eIF2*α*, thus promoting translational recovery. Collectively, the UPR relieves ER stress by decreased global protein synthesis, increased degradation of misfolded proteins, promoting chaperone synthesis, expansion of ER membrane volume, and triggering cell death [7].

### 2.1. Nutrient Excess, ER Stress, and Insulin Signaling

**Figure 1:**
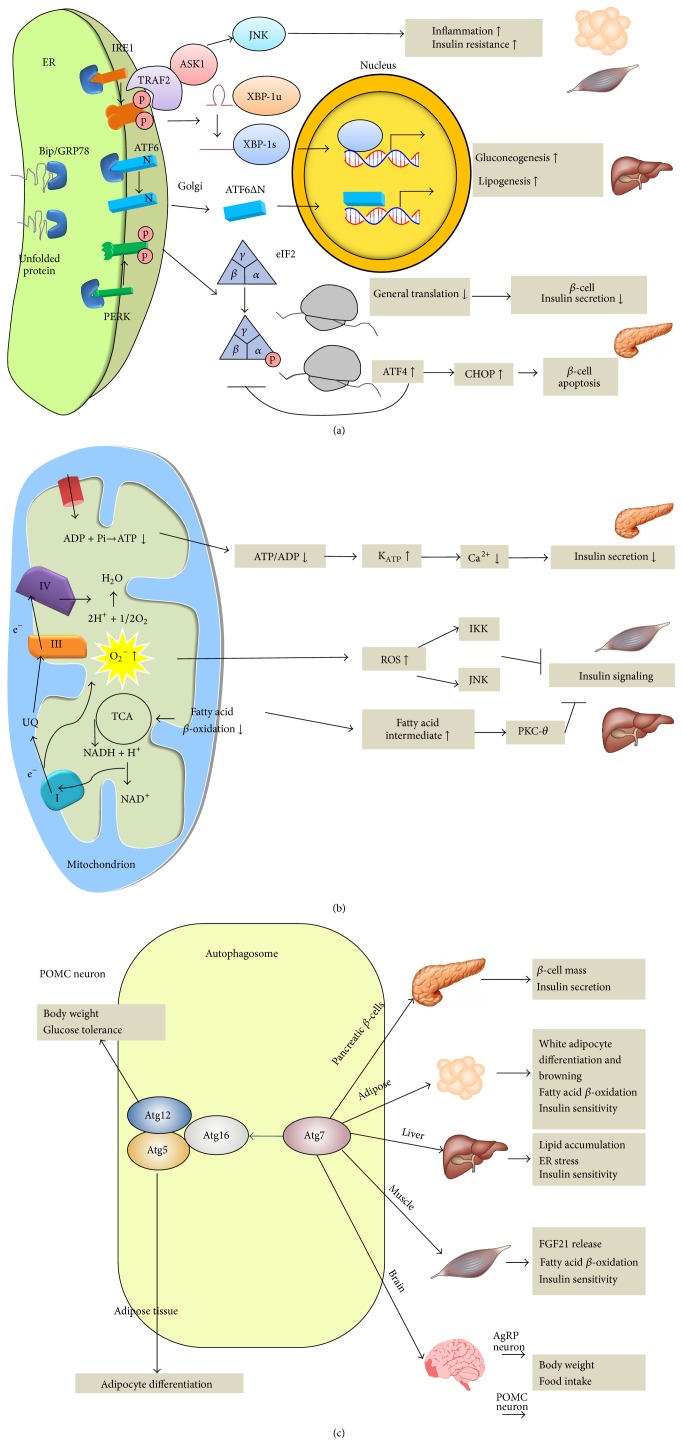
(a) Endoplasmic reticulum (ER) stress response and unfolded protein response (UPR) are linked to insulin resistance, inflammation lipogenesis, and pancreatic beta-cell survival. (b) Defective mitochondrial function leads to inflammation, insulin resistance, and reduced insulin secretion. (c) Autophagy regulates hepatic lipogenesis, adipocyte physiology, pancreatic beta-cell function, and appetite control. UPR: unfolded protein response; ROS: reactive oxygen species; NAD: nicotinamide adenine dinucleotide; NADH: reduced nicotinamide adenine dinucleotide; ADP: adenosine diphosphate; ATP: adenosine triphosphate; TCA: tricarboxylic acid cycle; K_ATP_: ATP-dependent potassium channel; UQ: ubiquinol; FGF21: fibroblast growth factor-21; AgRP: agouti-related peptide; POMC: proopiomelanocortin.

Several lines of evidence in human and mice indicate that chronic nutrient excess causes ER stress [8]. In contrast, ER stress is reduced by weight loss [9, 10]. Genetically manipulated mice models clearly demonstrate that ER stress and UPR influence insulin signaling and glucose homeostasis (Table 1, Figure 1(a)).* Xbp1* haploinsufficient mice show abnormal glucose intolerance and impaired insulin signaling in adipose tissue and liver on high-fat diet (HFD) [11]. The increased insulin resistance is mediated, at least in part, through IRE1-dependent activation of JNK. Conversely, hepatic overexpression of* Xbp1* lowers glucose in mice through interaction with FoxO1, a key transcriptional factor of gluconeogenesis [12], or uridine diphosphate (UDP) galactose-4-epimerase, an enzyme involved in galactose metabolism [13]. Mice with homozygous mutation at the eIF2*α* phosphorylation site (Ser51Ala) died at neonatal stage with defective gluconeogenesis [14]. Intriguingly, hepatic overexpression of* Gadd34,* which encodes an eIF2*α*-specific phosphatase that selectively counteracts PERK-eIF2*α* action, results in improved insulin sensitivity and diminished hepatic steatosis on HFD [15]. Hepatic overexpression of* Atf6* reduces gluconeogenesis [16] while silencing of hepatic* Atf6* increases gluconeogenesis [16]. The effect of ATF6 to suppress gluconeogenesis is mediated by disrupting the interaction between cAMP response element-binding protein (CREB) and transducer of regulated CREB protein 2 (TORC2), thereby decreasing the expression of gluconeogenic genes [16]. In addition, overexpression of chaperone GRP78 alleviates ER stress, restores insulin sensitivity, and resolves fatty liver in obese mice [17]. Similarly, deficiency of ER chaperone ORP150 results in impaired insulin signaling and impaired glucose tolerance, while overexpression of* Orp150* improves glucose tolerance and insulin signaling in obese mice [18]. These pieces of evidence strongly support that UPR modulates glucose homeostasis.

Mechanistically, all three canonical branches of UPR have been shown to promote inflammatory pathways. The activated IRE-1 recruits the tumor necrosis factor receptor associated factor 2 (TRAF2) and the apoptosis signal-regulating kinase 1 (ASK1) to the ER membrane, thereby activating JNK [19]. The PERK signaling has been shown to inhibit the translation of IKK*β*, the main negative regulator of NF-*κ*B, through phosphorylation of eIF2*α* [20]. ATF6 has also been shown to activate the NF-*κ*B pathway [21]. Both NF-*κ*B and JNK pathways are critical mediators of inflammatory response that impairs insulin signaling by serine phosphorylation of IRS1.

### 2.2. ER Stress and Lipid Synthesis

In addition to glucose homeostasis, the three UPR branches also regulate lipid synthesis (Table 1, Figure 1(a)). Selective deletion of* Xbp-1s* in the liver resulted in marked diminished hepatic cholesterol and triglyceride secretion and hepatic lipogenesis by downregulating genes involved in fatty acid synthesis [22], whereas liver-specific overexpression of* Xbp-1s* increases hepatic triglycerides content [13]. Targeted deletion of* Perk* in mammary gland inhibits lipogenic enzymes expression, resulting in reduced lipid content and milk production [23].* Atf6* knockout mice developed hepatic steatosis upon ER stress through regulation of genes involved in lipogenesis [24]. Similar phenotypes were observed in liver-specific* Ire1*-knockout mice and* eIF2α* loss-of-function mutation [25].

### 2.3. ER Stress and Insulin Secretion

Pancreas is exocrine and endocrine organ with heavy protein synthesis load. A transgenic green fluorescent mouse model for dynamic monitoring of ER stress detects significant ER stress signal (*Xbp1* mRNA splicing) in the pancreas 16 days after birth [26]. Several lines of evidence showed that UPR affect pancreatic islet survival and function (Table 1, Figure 1(a)). For example, mice with *β*-cell-specific deletion of* Xbp-1* displayed hyperglycemia and glucose intolerance resulting from decreased insulin secretion [27]. Translation attenuation through eIF2*α* phosphorylation prevents the oxidative stress and maintains the differentiated state of *β*-cells [28]. Preventing eIF2*α* phosphorylation in *β*-cells also causes hyperglycemia, indicating a significant role in PERK-eIF2*α* for islet survival [14].* Perk*-deficient mice develop severe hyperglycemia due to reduced islet mass [29, 30]. In human, a loss-of-function mutation in* Perk* causes a heritable form of juvenile diabetes (the Wolcott-Rallison syndrome) (Table 2), characterized by severe defects in pancreatic *β*-cells [31]. Furthermore, loss of CHOP, a downstream proapoptotic transcription factor of PERK-eIF2*α* arm, protects islets from apoptosis in the diabetic mice [32]. Hence, the two major pathological features of type 2 diabetes including peripheral insulin resistance and defective insulin secretion are both affected by ER stress and UPR.

## 3. The Role of Mitochondrial Dysfunction in Diabetes and Obesity

### 3.1. Mitochondrial Dysfunction and Insulin Resistance

Mitochondrion is a specialized organelle where tricarboxylic acid cycle, oxidative phosphorylation, and fatty acid *β*-oxidation occur. Reduced mitochondrial phosphorylation and fatty acid *β*-oxidation are consistently observed in skeletal muscle and liver of insulin-resistant human [33–35]. Furthermore, expression of genes involved in mitochondrial oxidative phosphorylation is coordinately reduced in insulin-resistant or type 2 diabetic subjects [36, 37]. Therefore, it is long hypothesized that, in the presence of excessive nutrient flux, defective mitochondria lead to increased superoxide production and fatty acid accumulation in skeletal muscle and liver, leading to insulin resistance.

In support of these findings, HFD has been shown to increase mitochondrial reactive oxygen species (ROS) emission and shift the cellular environment to oxidized state in muscle in mice and human [38–40]. Mitochondrion-targeted overexpression of catalase reduces mitochondrial ROS emission and prevents diet-induced insulin resistance in mice [38]. ROS has been shown to activate the proinflammatory JNK and through modulation of cysteine residue or IKK*β* [41–43], which in turn impairs insulin signaling via serine phosphorylation of IRS-1 (Figure 1(b)).

In addition to ROS, defective mitochondrial fatty acid *β*-oxidation leads to accumulation of triglycerides and fatty acids intermediates (e.g., diacylglycerol or ceramide) that activate PKC-*θ*, a serine/threonine kinase, thus attenuating insulin signaling [44, 45] (Figure 1(b)). Knockout of acetyl-CoA carboxylase 2 (*Acc2*), an enzyme generating malonyl-CoA which is a strong inhibitor of fatty acid oxidation, resulted in increased fatty acid oxidation, reduced adiposity, and improved insulin sensitivity [46]. Fat infusion increases fatty acids intermediates accumulation in muscle and induces insulin resistance in humans [47]. In contrast, pharmacological inhibition of ceramide (a fatty acid intermediate) production prevented fat-induced insulin resistance in mice and human [48] (Figure 1(b)).

However, whether the observed reduced mitochondrial function in insulin-resistant human is causative or compensatory for the development of insulin resistance is not certain in experimental mice model. Muscle- or liver-specific deletion of* Aif*, a mitochondrial protein essential for respiratory chain function, leads to decreased mitochondrial oxidative phosphorylation but improves insulin sensitivity [49]. Knockout of peroxisome proliferator-activated receptor-gamma coactivator 1-alpha (*Pgc1α*), a master regulator of mitochondrial biogenesis, resulted in decreased mitochondrial oxidative phosphorylation but protection from diet-induced obesity and insulin resistance in mice [50, 51]. Similarly, muscle- or adipose-specific knockout of the transcription factor A, mitochondria (*Tfam*), a key transcription factor for mitochondrial DNA transcription, causes abnormal mitochondrial morphology and function but improved glucose disposal [52, 53]. Furthermore, lower rate of fatty acid beta-oxidation and compromised mitochondrial oxidative phosphorylation caused by overexpression of the CDGSH iron sulfur domain 1 protein (*Cisd1*), which encodes an outer mitochondrial membrane protein blocking iron transport iron into the mitochondria, resulted in massive fat accumulation but improved insulin sensitivity [54] (Table 1). These data suggest that mitochondrial dysfunction does not cause insulin resistance.

From electrochemical point of view, mitochondrial superoxide (mostly from complex I) is generated when complex I is fully reduced with electrons but downstream electron transfer components are also fully reduced and thus cannot accept any more electrons (“electron jam”). In this situation, the saturated electrons in complex I leak and react prematurely with oxygen to form superoxide, a partially reduced form of molecular oxygen. This occurs when adenosine triphosphate (ATP) synthesis is not required or when the reduced nicotinamide adenine dinucleotide (NADH)/nicotinamide adenine dinucleotide (NAD^+^) ratio is high [55]. For mitochondria that are actively making ATP, the electrons are passed smoothly in the electron transfer train and hence the extent of superoxide production is low. When the ratio of NADH/NAD^+^ is low (such as diet restriction), complex I is not reduced so that electron leak is also low [55]. It is actually not certain whether reduced mitochondrial biogenesis or reduced oxidative phosphorylation rate by genetic manipulation would actually decrease or increase ROS production. This may explain the controversies between insulin resistance and various mitochondrial dysfunction models.

Another point of view, termed “mitohormesis” holds that increased ROS production from mitochondria may act as downstream effectors that trigger nuclear compensatory response including antioxidant defense and metabolic adaptation. An example comes from the observation that antioxidant treatment blocks the extension of life induced by nutrient deprivation in worm [56]. Mild mitochondrial stress appears to be beneficial for organism to adapt for subsequent metabolic perturbations [57].

### 3.2. Mitochondrial Dysfunction and Insulin Secretion

Mitochondrial ATP generation plays a pivotal role in insulin secretion of pancreatic *β*-cell. Increased mitochondrial ATP production in response to hyperglycemia closes the ATP-sensitive potassium channel, leading to membrane depolarization, opening of voltage-sensitive calcium channel, calcium ion influx, and insulin granule exocytosis (Figure 1(b)). Several forms of syndromic mitochondrial diseases are characterized with diabetes [58] (Table 2). Mutations in the mitochondrial DNA (mtDNA), especially those in tRNA genes such as A3243G mutation, cause approximately 0.5–1% of all types of diabetes [59, 60]. Consistently, *β*-cell-specific disruption of* Tfam* causes severe mtDNA depletion, deficient oxidative phosphorylation, abnormal appearing mitochondria in islets, and impaired insulin secretion [61]. Similarly, targeted disruption of frataxin, a mitochondrial iron-binding protein in pancreatic *β*-cell, causes increased islet ROS, decreased islet mass, and diabetes in mice [62]. Furthermore, patients with mutations in the frataxin gene develop diabetes in 23% of cases [63] (Table 2).

## 4. The Role of Autophagy in Diabetes and Obesity

Autophagy is a cellular housekeeping process which trafficked cytoplasmic misfolded protein and damaged organelles for lytic degradation and recycle, hence maintaining a normal cellular function [64]. During autophagy, part of the cytoplasm containing sequestered materials is bounded by a double membrane to form an autophagolysosome, which further fuses with lysosome for degradation. This process involves induction, cargo recognition, and nucleation that are tightly controlled by a group of over 30 autophagy-related (ATG) proteins [65].

Autophagy is originally considered as a protein turnover process to replenish amino acid pool during starvation. This signaling process is converged to the mammalian target of rapamycin complex 1 (mTORC1) pathway and is strongly affected by the nutrient level or growth factors such as insulin. During nutrient-rich condition, mTORC1 is activated to phosphorylate Atg1/UNC51-like kinase 1 (ULK-1) complex and inactivate the autophagy process. Conversely, during starvation, the adenosine monophosphate (AMP) to ATP increases. The energy depletion is sensed by AMP-activated protein kinase (AMPK) which activates autophagy by blocking mTORC1 activity and direct phosphorylation of Atg1/ULK1 [66]. A study using transgenic mouse model expressing a fluorescent marker of autophagy revealed that starvation activates autophagy in liver, heart, skeletal muscle, and kidney [67]. During starvation, autophagy provides amino acid for cellular fueling, protein synthesis, gluconeogenesis, and lipid mobilization.

**Figure 2:**
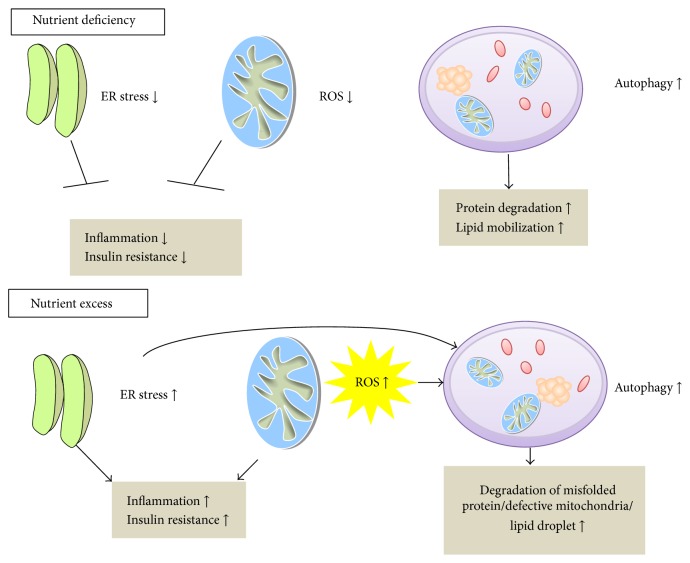
Interactions between endoplasmic reticulum (ER) stress, mitochondrial reactive oxygen species (ROS), and autophagy during nutrient deficiency and excess.

In stressful condition such as increased mitochondrial ROS, ER stress, or accumulation of excessive lipid droplet, autophagy is activated to degrade defective mitochondria (mitophagy), stressed ER (ER-phagy), or accumulated lipid (lipophagy) to remove excessive ROS, ER stress, or lipid [68] (Figure 2).

### 4.1. Autophagy and Hepatic Lipid Metabolism

Obesity is associated with downregulation of autophagy in the liver [69]. Autophagy of lipid droplet (lipophagy) in hepatocyte facilitates the degradation of lipid in the liver and defective autophagy leads to massive accumulation of triglyceride, ER stress, and insulin resistance in the liver [69, 70]. In contrast, restoration of the* Atg7* expression in liver resulted in alleviated ER stress and improved hepatic sensitivity in obese mice [69].

### 4.2. Autophagy and Insulin Secretion

Pancreatic *β*-cells keep on synthesizing large amount of insulin to maintain normoglycemia. When the protein folding cannot keep pace with the massive synthesis rate such as during hyperglycemia, UPR occurred to halt the process [71]. ER-phagy is the specific term for autophagic control to degrade excessive misfolded protein to the lysosome for degradation and prevent insulin secretory defects [72, 73]. Disruption of* Atg7* in pancreatic *β*-cells causes ER stress, reduction of *β*-cells mass, and increase in *β*-cells apoptosis [74, 75]. IAPP is another peptide hormone released from *β*-cells, which normally are cosecreted with insulin [76]. Intracellular oligomer accumulation of human islet amyloid polypeptide (hIAPP) is toxic to *β*-cells, which is a typical morphological change in T2DM. Abnormal hIAPP aggregates are primary degraded by autophagy. Transgenic mice expressing hIAPP with *β*-cell-specific* Atg7* deletion accumulate hIAPP oligomers and develop diabetes with increased oxidative damage and decreased *β*-cell mass [77–79] (Table 1, Figure 1(c)). Density volume of autophagic vacuoles and autophagosomes was significantly higher in *β*-cells of diabetic human [80].

Mitophagy also acts to prevent the accumulation of depolarized mitochondria and maintain optimal *β*-cells mitochondrial function [81]. *β*-cells-specific* Atg7* knockout mice showed swollen mitochondria and reduced insulin secretion (Table 1, Figure 1(c)) [75].

### 4.3. Autophagy, Adipose Tissue, and Skeletal Muscle

In contrast to the role of autophagy in hepatic lipid clearance and alleviating stress of pancreatic *β*-cells, the function of autophagy in adipose tissue and skeletal muscle deserves separate mention. Mice targeted with* Atg7* disruption in adipose tissue have reduced body fat, increased fatty acid *β*-oxidation, and improved insulin sensitivity [82, 83], indicating that autophagy is required for the production of large lipid droplets characteristic of white adipose tissue. However, *Atg*7^+/−^-*ob/ob* mice showed exacerbated insulin resistance with elevated lipid levels [84] (Table 1, Figure 1(c)). Muscle-specific* Atg7* knockout mice exhibit lean phenotype with increased lipolysis and *β*-oxidation rate in adipose tissue, enhanced glucose tolerance, and improved insulin sensitivity [85]. This is due to the impairment of autophagy to degrade defective mitochondria, which leads to the fibroblast growth factor (FGF21) release, causing lipolysis and *β*-oxidation rate in adipose tissue [85]. These diverse results of the same gene exerting different function in different organs may be a result of noncell autonomous function.

### 4.4. Autophagy and Appetite Control

Furthermore, food intake in mice with agouti-related peptide (AgRP) neuron-specific* Atg7* deletion was decreased while it increased in proopiomelanocortin (POMC) neuron-specific* Atg7* deletion. The changes of the functional consequences converge on the controlling of a common neuropeptide, *α*-melanocyte-stimulating hormone (*α*-MSH), level (Table 1, Figure 1(c)).

## 5. Targeting Organelle Stress for Treating Metabolic Diseases

Chemical chaperones including tauroursodeoxycholate (TUDCA) and 4-phenylbutyrate (PBA) have been shown to reduce ER stress and improve insulin sensitivity in rodents and human [86, 87] (Table 3). These two drugs have been approved by the US Food and Drug Administration for the treatment of primary biliary cirrhosis. Numerous small molecules are identified to increase chaperone expression or to modulate specific arm of UPR* in vitro* using various screening strategies. For example, GSK2606414 has been shown to inhibit PERK kinase activity [88], azoramide to activate ATF6 [89], valproate to increase GRP78 expression [90], salubrinal and guanabenz to inhibit eIF2*α* dephosphorylation [91, 92], and 3-ethoxy-5,6-dibromosalicylaldehyde [93], STF-083010 [94], MKC-3946 [95], 4*μ*8C [96], and KIRA6 to inhibit IRE1 RNase activity [97]. Among them, valproate has been shown to attenuate atherosclerosis and alleviate hepatic steatosis [90] and azoramide has been shown to improve insulin sensitivity and pancreatic *β*-cell function in rodent models [89].

Pharmacological approaches to alleviate mitochondrial stress include carnitine [98, 99], Coenzyme Q_10_ [100], ROS scavengers (peptide SS31 [38], *α*-lipoic acid [101–110], vitamin E, beta-carotene, vitamin C [111–114],* N*-acetylcysteine [115–118], and mitoQ [119]), stimulators of mitochondrial biogenesis (resveratrol and other sirtuin activators [120], and estrogen-related receptor modulators [121]). Specifically, carnitine or carnitine-orotate complex, which promotes fatty acid *β*-oxidation, improves insulin sensitivity or attenuates hepatic steatosis in most randomized clinical trials [98, 99]. Coenzyme Q1, however, showed no net effect on glycemic control in most type 2 diabetic patients [100]. Most evidence demonstrated that *α*-lipoic acid is a potent weight-reducing and insulin sensitizing agent in human clinical trials and rodent models [101–110]. Multiple small clinical trials investigating the effect of antioxidant vitamin E, vitamin C, and beta-carotene on glycemic control in diabetic patients yielded inconsistent results [111–115]. However, in a randomized clinical trial of 247 adults with nonalcoholic steatohepatitis, vitamin E use, as compared with placebo, was associated with a significantly higher rate of improvement in nonalcoholic steatohepatitis [122]. N-acetylcysteine, an approved drug for acetaminophen intoxication and mucolysis, has been demonstrated to prevent diet-induced obesity in rodent models [116–118]. Significant controversies remained regarding the metabolic action of resveratrol in human; a meta-analysis of 11 randomized controlled trials revealed that resveratrol significantly reduces glucose, insulin, and insulin resistance in diabetic patients but not in nondiabetics [120] (Table 3). Further results from clinical trials and more potent SIRT1-activating compounds (STAC) such as SRT1720 and SRT2104 are awaited.

Various therapeutic agents may be used to enhance autophagy. Trehalose is an autophagy enhancer which improves the glucose intolerance of hIAPP transgenic mice fed a HFD and further reduced hIAPP oligomer accumulation and improved *β*-cells function [77]. Both Imatinib and trehalose were reported to improve metabolic parameters of *Atg*7^−/−^ *-ob/ob* mice by enhanced autophagic flux [84]. Dihydro-CDDO-trifluoroethyl amide (dh404) is an Nrf2 activator which can reduce oxidative stress in isolated rat islet by enhancing autophagy [123] (Table 3).

## 6. Future Perspectives

The interaction between ER stress, mitochondrial oxidative stress, and autophagy is complex. Most small molecules used to date do not have the required specificity. Furthermore, the multiple intrinsic feedback pathways, the cross-organ communication, and the interplay between autophagy and carcinogenesis make it difficult to target a single pathway to treat metabolic diseases without triggering unwanted side effects. Currently, the most efficient and safe way to reduce organelle stress and to treat metabolic disease is probably prevention of overnutrition.

## Figures and Tables

**Table 1:** Genetically modified mice model linking organelle stress to metabolic diseases.

Model	Gene function	Tissue	Phenotypes
*Xbp1*	UPR	Global haploinsufficiency	Weight gain, glucose intolerance, and insulin resistance on HFD [11]
*Xbp1*	UPR	Liver-specific KO	Diminished hepatic cholesterol and triglyceride secretion and hepatic lipogenesis [22]
*Xbp1*	UPR	Liver-specific OE	Reducing serum glucose concentrations and increasing glucose tolerance [12] Fasting and postprandial hypoglycemia; increased hepatic triglyceride content [13]
*Xbp1*	UPR	*β*-cell-specific KO	Hyperglycemia and glucose intolerance resulting from decreased insulin secretion [14]
*Perk*	UPR	Mammary epithelium-specific KO	Reduced accumulation of lipid content and the milk produced [23]
*Perk*	UPR	*β*-cell-specific KO	Hyperglycemia associated with loss of islet and *β*-cell architecture [29, 30]
*eIF2α*	UPR	Phosphorylation site mutation	Defective gluconeogenesis and deficiency of pancreatic beta-cell [14]
*Gadd34*	UPR	Liver-specific OE	Lower liver glycogen levels, fasting hypoglycemia, diminished hepatics steatosis [15]
*Atf6*	UPR	Liver-specific OE/silencing	Increased hepatic glucose output/lowered hepatic glucose output [16]
*Atf6*	UPR	Global KO	Hepatic steatosis [24]
*Atf6, eIF2α, Ire1*	UPR	Global KO/phosphorylation site mutation	Hepatic steatosis [25]
*Chop*	UPR	Global KO	Delayed the onset of diabetes and beta-cell apoptosis [32]
*Grp78*	Chaperone	Liver-specific OE	Reduced hepatic triglyceride and cholesterol contents and improved insulin sensitivity improved [17]
*Orp150*	Chaperone	Liver-specific OE/Silencing	Improved insulin resistance and ameliorated glucose tolerance/increased insulin resistance [18]
*Aif*	Mitochondrion-localized flavoprotein	Muscle and liver-specific KO	Improved glucose tolerance, reduced fat mass, and increased insulin sensitivity [49]
*Pgc-1α*	Mitochondrial biogenesis	Global KO	Resistance to diet-induced obesity and insulin resistance [50, 51]
*Tfam*	Mitochondrial DNA transcription	Muscle-specific and adipose-specific KO	Improved glucose disposal [52, 53]
*Tfam*	Mitochondrial DNA transcription	*β*-cell-specific KO	Reduced *β*-cell mass and insulin secretion [61]
*Cisd1*	Mitochondrial iron transport	Global and liver-specific OE	Massive expansion of adipose tissue but improved insulin sensitivity [54]
*Fxn*	Assembly of iron-sulfur cluster in mitochondria	*β*-cell-specific KO	Increased islet oxidative stress, reduced islet mass, and diabetes [62]
*Atg5*	Autophagy	Adipose-specific KO	Impaired adipocyte differentiation [124]
*Atg5*	Autophagy	Global OE	Lean, enhanced glucose tolerance, insulin sensitivity, and extended lifespan [125]
*Atg7*	Autophagy	Global KO	Increased hepatic ER stress and impaired insulin sensitivity [69]
*Atg7*	Autophagy	*β*-cell-specific KO	Reduction of *β*-cells mass, reduced insulin secretion, mitochondria swelling, and lower ATP production [74, 75]
*Atg7*	Autophagy	Adipose-specific KO	Lean, browning of white adipose tissue, increased fatty acid oxidation, and improved insulin sensitivity [82, 83]
*Atg7*	Autophagy	Muscle-specific KO	Reduced weight and body fat, enhanced glucose tolerance and insulin sensitivity, enhanced lipolysis and fatty acid oxidation, and increased FGF21 level [85]
*Atg7*	Autophagy	AgRP neuron-specific KO	Lean with decreased food intake [126]
*Atg7*	Autophagy	POMC neuron-specific KO	Increased body weight and food intake, impaired glucose tolerance [127, 128]
*Atg7*	Autophagy	*Myf*5+ progenitors-specific KO	Impaired brown adipose tissue and skeletal muscle differentiation, browning of white adipose tissue, increased energy expenditure, increased body temperature, impaired glucose tolerance [129]
*Atg7*	Autophagy	*β*-cell-specific KO in hIAPP transgenics	Decreased *β*-cell mass and diabetes [77–79]
*Atg7*	Autophagy	Global haploinsufficiency in *ob/ob* mice	Reduces ER stress; improves insulin sensitivity and glucose tolerance *ob/ob* mice [84]
*Atg7*	Autophagy	Liver-specific OE in *ob/ob* mice	Improved insulin sensitivity and glucose tolerance [69]
*Atg12*	Autophagy	POMC neuron-specific KO	Weight gain, adiposity, and impaired glucose tolerance under HFD [130]

KO: knockout; OE: overexpression; UPR: unfolded protein response; HFD: high-fat diet; AgRP: agouti-related peptide; POMC: proopiomelanocortin; hIAPP: human islet amyloid polypeptide.

**Table 2:** Human hereditary syndrome linking organelle stress and diabetes mellitus.

Disease	Gene	Function	Phenotypes
Wolcott-Rallison syndrome	*PERK*	UPR	Neonatal or early-infancy diabetes, epiphyseal dysplasia, osteoporosis, and growth retardation [31]
Wolfram syndrome	*WFS1*	Negative regulator of UPR	Neurological dysfunctions and diabetes [131]
Friedreich's ataxia	*FXN*	Assembly of iron-sulfur cluster in mitochondria	Ataxia, cardiac dysfunction, and diabetes [63]
Kearns-Sayre syndrome	Large deletion of mitochondrial DNA	Respiratory chain	Ataxia, weakness, ptosis, pigmentary retinopathy, and diabetes [58]
MELAS (Mitochondrial encephalomyopathy, lactic acidosis, and stroke-like episodes)	Mitochondrial tRNA	tRNA	Seizure, ataxia, hemiparesis, cortical blindness, diabetes, and short stature [58]

**Table 3:** Treatment targeting organelle stress for diabetes mellitus and obesity.

Agent	Specific mechanism	Highest level of studies	Result
Tauroursodeoxycholic acid	Chemical chaperone	Randomized controlled trials	Improved insulin sensitivity in muscle and liver in obese individuals [86]
Phenylbutyrate	Chemical chaperone	Randomized controlled trials	Improved insulin sensitivity and beta-cell function in lipid-infused individuals [87]
Azoramide	ATF6 activators	Rodents	Improves insulin sensitivity and beta-cell function in obese mice [89]
Valproate	Increasing GRPP78	Rodents	Ameliorates atherosclerosis and hepatic steatosis in *Apoe* ^−/−^mice [90]
L-Carnitine or carnitine-orotate	Fatty acid transfer for beta-oxidation	Randomized controlled trials	Twelve of 17 studies showing improved insulin sensitivity or glycemic control in type 2 diabetic patients or alleviation of hepatic steatosis [98, 99]
Co-enzyme Q_10_	Electron carrier from complex I and II to complex III	Randomized controlled trials	No net effect on glycemic control in type 2 diabetic patients [100]
*α*-lipoic acid	Antioxidant	Randomized controlled trials; rodent	Weight-reducing, glucose-lowering, and insulin-sensitizing effect; prevention of hepatic steatosis [101–110]
Vitamin E	Antioxidant	Randomized controlled trials	Inconsistent results on glycemic control [111–115]; reduced hepatic steatosis [122]
*N*-acetylcysteine	Antioxidant	Rodents	Prevents diet-induced obesity [116–118]
Peptide SS31	Mitochondria-targeted antioxidant peptide	Rodent	Improved glucose tolerance in diet-induced obese mice [38]
Resveratrol	SIRT1 agonist	Randomized controlled trials	Improved insulin sensitivity and glycemic control in diabetic patients; no effect in nondiabetic patients [120];
GSK5182	Estrogen-related receptor gamma inverse agonist	Rodents	Reduces hyperglycemia due to inhibition of hepatic gluconeogenesis [121]
Trehalose, imanitib	Enhance autophagy	Rodents	Improved glucose tolerance and insulin sensitivity in obese mice [84]
Dh404	Nrf2 activator	Rodents	Increased viability of islet by enhancing autophagy [123]
